# PreAnaesThesia computerized health (PATCH) assessment: development and validation

**DOI:** 10.1186/s12871-020-01202-8

**Published:** 2020-11-14

**Authors:** Tarig Osman, Eileen Lew, Elaine Pooi-Ming Lum, Louise van Galen, Rajive Dabas, Ban Leong Sng, Josip Car

**Affiliations:** 1 Centre for Population Health Sciences, Lee Kong Chian School of Medicine, Nanyang Technological University (Singapore), Singapore, Singapore; 2 Department of Women’s Anaesthesia, KK Women’s and Children’s Hospital (Singapore), 100 Bukit Timah Road, Singapore, 229899 Singapore; 3 Present address: Duke-NUS Medical School, 8, College Road, Singapore, 169857 Singapore; 4 Present address: Department of Internal Medicine, VU University Medical Center, De Boelelaan 1117, Room ZH 4A58, 1081 HV Amsterdam, Netherlands; 5 Global eHealth Unit, Department of Primary Care and Public Health, School of Public Health, Imperial College London (United Kingdom), London, UK

**Keywords:** Preanaesthesia assessment, Computer-assisted history-taking system, Digital health

## Abstract

**Background:**

Technological advances in healthcare have enabled patients to participate in digital self-assessment, with reported benefits of enhanced healthcare efficiency and self-efficacy. This report describes the design and validation of a patient-administered preanaesthesia health assessment digital application for gathering medical history relevant to preanaesthesia assessment. Effective preoperative evaluation allows for timely optimization of medical conditions and reduces case cancellations on day of surgery.

**Methods:**

Using an iterative mixed-methods approach of literature review, surveys and panel consensus, the study sought to develop and validate a digitized preanaesthesia health assessment questionnaire in terms of face and criterion validity. A total of 228 patients were enrolled at the preoperative evaluation clinic of a tertiary women’s hospital. Inclusion criteria include: age  ≥ 21 years, scheduled for same-day-admission surgery, literacy in English and willingness to use a digital device. Patient perception of the digitized application was also evaluated using the QQ10 questionnaire. Reliability of health assessment questionnaire was evaluated by comparing the percentage agreement of patient responses with nurse assessment.

**Results:**

Moderate to good criterion validity was obtained in 81.1 and 83.8% of questions for the paper and digital questionnaires respectively. Of total 3626 response-pairs obtained, there were 3405 (93.4%) concordant and 221 (6.1%) discrepant response-pairs for the digital questionnaire. Discrepant response-pairs, such as ““no/yes*”* and “unsure/yes*”*, constitute only 3.7% of total response-pairs. Patient acceptability of the digitized assessment was high, with QQ10 value and burden scores of 76 and 30%, respectively.

**Conclusions:**

Self-administration of digitized preanaesthesia health assessment is acceptable to patients and reliable in eliciting medical history. Further iteration should focus on improving reliability of the digital tool, adapting it for use in other languages and incorporating clinical decision tools.

**Supplementary Information:**

The online version contains supplementary material available at 10.1186/s12871-020-01202-8.

## Background

Current practice guidelines mandate that patients undergo preanaesthesia assessment prior to surgery and anaesthesia, defined as the process of clinical assessment that precedes the delivery of anaesthesia care for surgery and non-surgical procedures [1]. Its goal is to allow for timely identification and optimization of medical conditions, thereby reducing perioperative morbidity and mortality. Effective preoperative evaluation can also decrease case delays and cancellations on day of surgery [2].

Traditionally, preanaesthesia assessment is conducted by a health care provider via a face-to-face interview with the patient. Studies suggest that self-administration of digital assessment questionnaires is a feasible means of gathering medical information for preanaesthesia assessment [3–12]. Compared with in-person interviews, these digital self-assessment tools are associated with patient acceptance and satisfaction, reliability of information and improved efficiency of assessment [4, 6, 9].

At the preoperative evaluation clinic of our hospital, a 33-item preanaesthesia health assessment paper questionnaire is currently administered to elective surgical patients by nurses to gather medical information pertinent to preanaesthesia assessment [13]. Based on pre-determined criteria, responses help to identify patients with medical issues who require outpatient anaesthetic review 2 to 4 weeks in advance of surgery. Relatively healthy patients are allowed to bypass outpatient referral and undergo standard anaesthetic review on the day of surgery. The questionnaire has served our purpose well, but is not designed for patient self-administration as it contained technical language and medical terms.

In line with global advances in information technology, healthcare institutions are increasingly leveraging on digital health technologies for care delivery. Local hospital statistics indicate that an average 900 patients are scheduled for elective surgeries every month and this number is expected to increase, as disease burden increases with an aging population. To cope with this demand, we postulate that a patient self-administered digital health assessment tool can be developed and implemented for the purpose of gathering medical history relevant for preanaesthesia assessment. The virtual tool allows remote access, so that assessment questionnaires can be completed at a time, place and pace convenient to the patient. The present study describes our experience in the development and validation of a patient-administered digital preanaesthesia health assessment questionnaire on a tablet device at a tertiary hospital.

## Methods

The study was conducted at a preoperative evaluation clinic that provides care for women scheduled for elective surgery at a tertiary hospital. A working group comprising three consultant anaesthetists, six clinic nurses and five digital health researchers from a local medical school sought to develop a patient self-administered digital preanaesthesia health assessment application through an iterative process. Ethics approval was granted by the Nanyang Technological University Institutional Review Board (Ref: IRB-2017-12-011) and the SingHealth Institutional Review Board (Ref: 2017/3002).

**Fig. 1:**
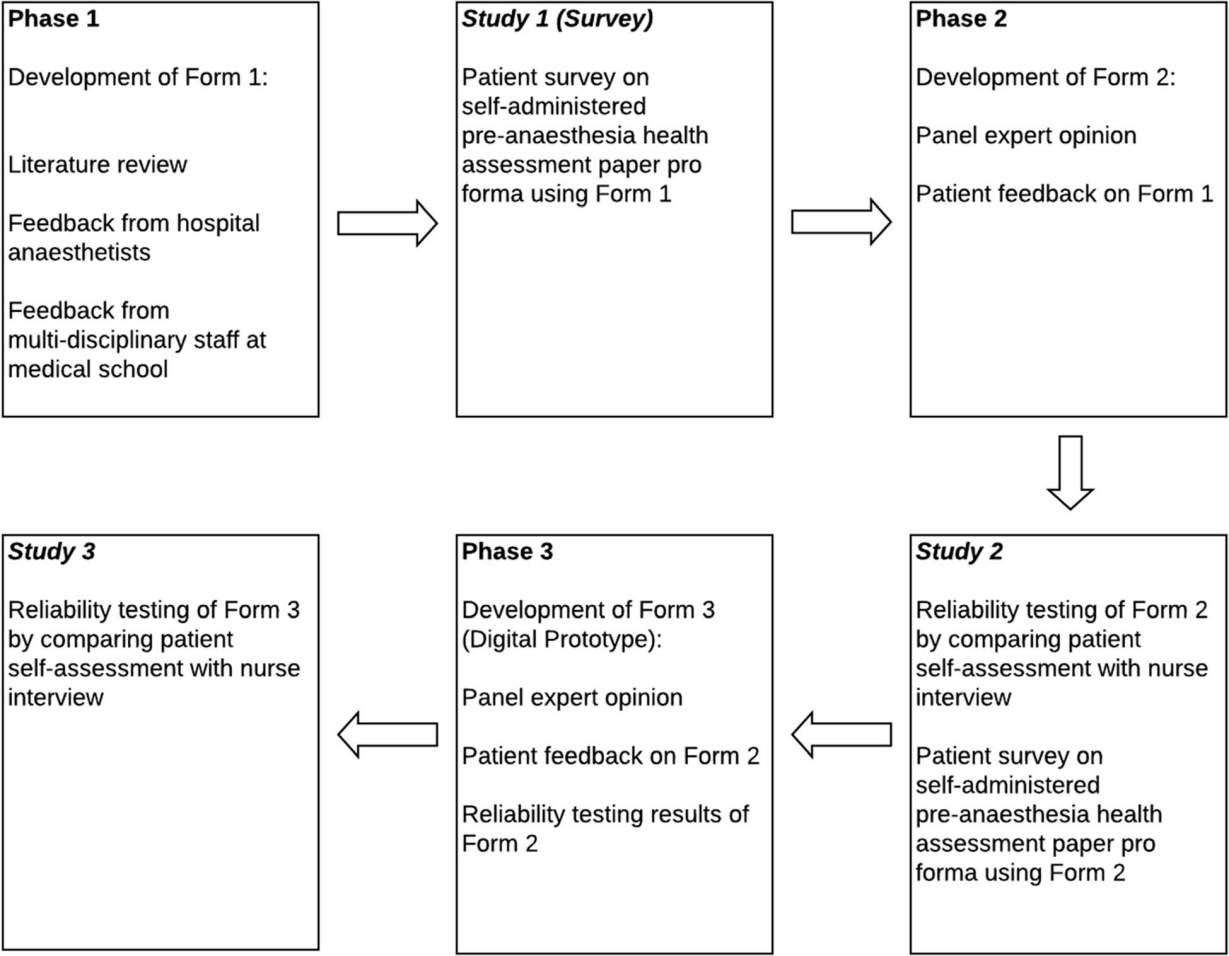
Mixed-methods approach to the development of PATCH

A mixed-methods approach was adopted. A paper version of the questionnaire was first designed and validated, before conversion to a digital prototype. We hereby refer to the paper versions as Forms 1 and 2 and the digital version as Form 3. All versions of the questionnaire were developed in English and iteratively, each version was an improvement over the previous. Figure 1 describes the phases of development and validation of the questionnaires from paper to digital formats.

### Phase 1: development and assessment of form 1

The self-administered paper questionnaire, Form 1, was designed after an extensive review of relevant literature via Pubmed and Google Scholar. Search terms used include (*preanaesthesia* or *preanesthesia* or *pre-anaesthetic* or *pre-anesthetic* or *preoperative* or *pre-operative*) and *(health assessment* or *screening* or *questionnaire)* and*/*or *(validation)*. Shortlisted questionnaires were further examined for scope and relevance of domains and items, options of response types (i.e. binary/non-binary/free-text response), and design format of questions. Through consensus, the working group also determined the clinically relevant domains and corresponding items to be included in Form 1.

First draft of Form 1 was then presented to twelve attending anaesthetists of the hospital for evaluation of its face validity. While the domains were deemed adequate, the anaesthetists suggested the addition of follow-up questions to qualify some items e.g. number of pack-years as a follow-up to a positive history of cigarette smoking.

The draft of Form 1 was also evaluated at a workshop, where multidisciplinary staff of a local medical school provided feedback on its readability, clarity and contextualization. Participants suggested terms to replace technical jargon and reduced ambiguity in questions. Questions were structured according to domains of the body systems and each question was verified to assess only one domain or concept to the extent permissible (Supplementary Box 1). A glossary of terms (Supplementary Box 2) was also appended to provide explanation of medical terms.

With the collective feedback obtained, Form 1 was finalised and administered as a pen-and-paper survey to a convenience sample of 33 patients in a pilot study, herewith referred to as “Study 1”. The aim of this survey was to identify problems that were not addressed or considered during the design of the questionnaire. Inclusion criteria for patient recruitment were: age ≥ 21 years, ability to read and write in English and scheduled for same-day-admission surgery. After written informed consent, all patients were given instructions on the completion of Form 1, with emphasis on unassisted self-assessment. Following that, each patient underwent a semi-structured interview using questions adapted from the QQ-10 questionnaire, [14] an established instrument for measuring face validity, feasibility and utility of healthcare questionnaires. For our purpose, the original QQ-10 questionnaire was modified by amending options of “mostly disagree*”* to “disagree*”* and “mostly agree*”* to “agree”. Upon completion of the interview, each patient underwent a nurse-led assessment as per standard of care. Demographic data and time taken to complete Form 1 were recorded. Data from Form 1 was analyzed using IBM SPSS version 25 (IBM corp. Armonk, NY, USA). Data from the QQ-10 questionnaire was analyzed both quantitatively and qualitatively, using thematic analysis.

### Phase 2 – iteration of form 1 to form 2, with validation

Form 2 was an iteration of Form 1, based on the feedback received from participants of Study 1. Improvements included the re-phrasing of questions to improve clarity and insertion of visual illustrations. The explanation of eight terms in the glossary section was also edited to improve ease of understanding (Supplementary Box 3).

A validation study targeting a larger convenience sample size of 104 patients was conducted. Referred as “Study 2”, 104 patients scheduled for same-day-admission surgery were recruited on presentation to the preoperative evaluation clinic during the designated study period. The primary aim of the study was to evaluate the criterion validity of Form 2 before its conversion to a digital prototype. The inclusion criteria were similar to those of Study 1.

The sample size was chosen, based on a similar study reported in the literature [15]. Consenting patients first completed Form 2 independently, after which their responses were verified by the nurse via a structured face-to-face interview, guided by Form 2. If a discrepancy of response was noted, the nurse would make annotations upon verification with the patient. Each patient was also interviewed using the modified QQ-10 questionnaire, as described for Study 1.

Criterion validity of the questionnaire was assessed by measuring the agreement between the patient responses and those obtained during nurse assessment. To account for questions with prevalence < 5% or > 95%, we have opted to report percentage agreement (PA), instead of the Kappa coefficient. PA is defined as number of questions with concurring responses divided by the total number of questions. Criterion validity is considered good if PA ≥ 95%, moderate if PA between 90 to < 95% and poor when PA < 90% [10]. The frequency of identical (“Yes/Yes*”*, *“*No/No*”* and *“*Unsure/Unsure*”*), contradictory (“Yes/No*”* or “No/Yes”), and non-contradictory (“Unsure/Yes*”*, “Unsure/No*,”* “Yes/Unsure*”,* and “No/Unsure*”*) response pairs were also analysed. Sum of the contradictory and non-contradictory response rates describe the total discrepancy error rate. Data were analyzed using IBM SPSS version 25, as described for Survey 1.

### Phase 3 – development and validation of form 3

The iteration of Form 2 to Form 3 (the first digital protoype) was based on findings obtained from Phase 2 and renewed input from the working group (Supplementary Box 4). The digital application, called PreAnaesThesia Computerized Health assessment, or PATCH, was developed on an iOS platform on a tablet, using React Native (JavaScript framework). The server was made using NodeJS, a JavaScript framework. Data was stored on MongoDB database. For the purpose of the study, the server program and database were located on a secure server at the Nanyang Technological University.

Improvements adopted for Form 3 included further rephrasing of questions to reduce ambiguity and deletion of questions deemed to be irrelevant. To facilitate patients in listing their medications and previous surgeries, a drop-down list of common medications and surgeries was developed, using data gathered from participants in Phase 1 and 2. The glossary of terms was configured to appear as pop-up boxes of explanation when activated by screen-touch. In addition, the application was designed to provide a summary page for review and final edit before submission. Screenshots of the digital prototype are shown in Supplementary Figure 1.

As the criterion validity of a paper questionnaire does not necessarily extend to its electronic format [16], validation of the digital prototype, Form 3, was conducted in Study 3. In addition to the inclusion criteria described in the earlier phases, the ability to use a tablet device was added as a criterion for recruitment. One hundred and six patients were recruited at the preoperative evaluation clinic over 8 weeks. Consenting participants completed digital self-assessment on a tablet unaided, then underwent nurse assessment using a provider interface of the digital tool and with the nurse blinded to the patient’s responses. PA for each response pair was measured. Time to completion of self-assessment was automatically captured by the application. Data was analysed using IBM SPSS verison 25.

## Results

### Study 1 (survey)

Of 33 patients recruited, 32 completed the study. One patient was excluded when the nature of admission was converted from same-day-admission to inpatient. Patients identified themselves as Chinese (23/71.9%), Malay (4/12.5%), Indian (1/3.1%), and others (4/12.5%), consistent with the ethnic distribution in the local general population. Median (IQR) age was 37 (32.2, 43) years. Median (IQR) time to complete self-assessment was 4 (3, 5) minutes. None of the patients identified any question as being uncomfortable to answer (Table 1). Table 2 describes the patient perception of selected statements from the QQ-10. Overall, patient perception of self-assessment was favourable.

**Table 1:** Patients’ assessment of Form 1 (*n* = 32)

	Yes (n/%)	No (n/%)
**Relevance**		
Do you understand why we have asked you to complete the questionnaire?	32 (100)	0 (0)
Did the questions seem relevant to you and your medical history?	31 (96.9)	1 (3.1)
Language and content		
Did you understand most of the wording of the questionnaire?	31 (96.9)	1 (3.1)
Were there any medical terms you did not understand?	9 (28.1)	23 (71.9)
Were there any questions you felt were important but missed?	5 (15.6)	27 (84.4)
Did the questions prompt you to remember anything?	3 (9.4)	29 (90.6)
Was there any area that had too many questions on?	2 (6)	30 (94)
Were there any questions you did not feel comfortable/expect answering?	0 (0)	32 (00)

**Table 2:** Patient feedback on Form 1, based on modified QQ-10 questionnaire (n = 32)

	Strongly agree and agree n (%)	Neutral (n/%)	Strongly disagree and disagree (n/%)
The questionnaire was relevant to my condition.	30 (93.8)	0 (0)	2 (6.3)
The questionnaire was easy to complete.	30 (93.8)	0 (0)	2 (6.3)
I would be happy to complete it again in the future as part of my routine care.	25 (78.1)	5 (16)	2 (6)
The questionnaire was too embarrassing.	0 (0)	1 (3)	31 (97)
The questionnaire was too complicated.	3 (9)	4 (13)	25 (78)
The questionnaire was too long.	3 (9)	8 (25)	21 (66)

A total of 48 feedback comments were obtained from 21 patients. They pertained mostly to the clarification of medical terms (13/61.9%) and availability of options to guide entry of medications and past surgeries (8 / 38.1%). These comments were taken into consideration in the iteration of Form 1 to Form 2.

### Study 2

Of 104 patients recruited, 98 patients (94.2%) completed the study and 6 were excluded due to incomplete paperwork. The patients identified themselves as Chinese (50/51%), Malay (24/24.5%), Indian (10/10.2%), and others (14/14.3%), with a median (IQR) age of 38.5 (33, 44) years. Patients took 7.3 (5.6, 9.4) [median (IQR)] minutes to complete pre-anaesthesia self-assessment and generally responded favourably to statements measuring value in the QQ-10 questionnaire (Table 3). Among negative perceptions, length of questions emerged as the most frequent reason. Of note, 82 (83.7%) of participants were willing to utilize a digital version of the questionnaire in the future.

**Table 3:** Patient feedback on Form 2, based on modified QQ-10 questionnaire (*n* = 98)

	Strongly agree – agree (n/%)	Neutral (n/%)	Strongly disagree – disagree (n/%)
The questionnaire helped me to communicate about my condition with the nurse.	95 (96.9)	2 (2.04)	1 (1.02)
The questionnaire was easy to complete.	89 (90.8)	8 (8.2)	1 (1.02)
The questionnaire included all the aspects of my condition that I am concerned about.	88 (89.8)	9 (9.2)	1 (1.02)
The questionnaire was relevant to my condition.	85 (86.7)	12 (12.2)	1 (1.02)
I would be happy to complete it again in the future as part of my routine care.	75 (76.5)	16 (16.3)	7 (7.1)
I enjoyed filling in the questionnaire.	56 (57.1)	32 (32.7)	10 (10.2)
The questionnaire was too long.	15 (15.3)	36 (36.7)	47 (48)
The questionnaire was too complicated.	7 (7.1)	19 (19.4)	72 (73.5)
The questionnaire was too embarrassing.	1 (1.02)	9 (9.2)	88 (89.8)
The questionnaire upset me.	2 (2)	6 (6.1)	90 (91.8)
The information sheet was helpful.	89 (90.8)	8 (8.2)	1 (1)
If you had to complete this at home or in the clinic online, do you think you could?	77 (78.6)	16 (16.3)	5 (5.1)
I liked completing the questionnaire while in the waiting area.	65 (66.3)	21 (21.4)	12 (12.2)
I am comfortable answering sensitive questions in the questionnaire first than I would with the nurse.	70 (71.4)	20 (20.4)	8 (8.2)
I answered the questionnaire truthfully to the best of my knowledge.	94 (95.9)	4 (4.1)	0 (0)
I am willing to take an iPad version of this questionnaire in the future.	82 (83.7)	13 (13.3)	3 (3)
I prefer to talk to the nurse/doctor instead completing the questionnaire.	25 (25.5)	43 (43.9)	30 (31)

Analysis of patient feedback on the design of Form 2 revealed a total of 56 comments from 32 (32.7%) patients. Majority of comments referred to the need for clarification of medical terms (23/71.9%). There were requests to shorten the length of the questionnaire (3/9.4%). Overall QQ-10 value and burden scores were 76% (SD = 13%) and 30% (SD = 12.5%), respectively. Mean score for value questions ranged from 2.9 to 3.3, while the mean score for burden questions ranged from 0.9 to 1.64.

Table 4 shows the inter-rater reliability of Form 2. Good criterion validity was attained for 24 of 37 (65%) questions. Six (16%) questions were classified as having moderate criterion validity while seven (19%) had poor criterion validity. Total number of response pairs was 3626. Of these, 3432 were identical, giving a concordance rate of 94.6%. Sixty-seven (1.8%) were discrepant contradictory responses while 127 (3.5%) were discrepant non-contradictory responses, giving total discrepant responses of 194 (5.4%). Of these, the most common discrepant response pair was “unsure/no*”* (94/2.6%), followed by “yes/no*”* (63/1.7%) and “unsure/yes*”* (32/0.9%).

**Table 4:** Inter-rater Reliability Testing of Form 2

		PA	Criterion validity
1	Do you have any allergies (to medicines, sticking plaster, iodine, latex, food, etc.)?	94	Moderate
2	As medicines and supplements can affect body functions and interact with anaesthetics, please list all the medicines (including traditional medicines and health supplements) you are currently taking on a regular or daily basis in the last 2 weeks. ^a^	–	–
3	Have you ever had an operation?	97	Good
4	Are you ever short of breath after walking up two flights of stairs or an overhead bridge?	88	Poor
5	Was your heart activity ever measured using wires on your chest (an ECG or electrocardiogram)?	76	Poor
6	Has a doctor ever told you, you have high blood pressure, also known as ‘hypertension’?	96	Good
7	Do you have, or have you ever had chest pain that you felt tight or heavy (not from coughing)?	88	Poor
8	Have you ever had a heart attack?	100	Good
9	Do you have frequent swelling in feet or ankles?	89	Poor
10	Do you have, or have you ever had treatment for problems with your heartbeat (too low, too fast, irregular)?	91	Moderate
11	Has a doctor ever told you they heard an abnormal sound (e.g. a click or a murmur) whilst listening to your heart?	98	Good
12	Do you have a cardiac pacemaker or an implanted cardioverter-defibrillator?	100	Good
13	Have you ever had heart surgery (valve or stent or bypass operation)?	99	Good
14	Do you have or have you ever had blood clots in legs or lungs?	98	Good
15	Have you ever had a blood transfusion?	99	Good
16	Do you have asthma or have you had asthma as a child?	98	Good
17	Do you currently have a cough lasting more than 8 weeks?	99	Good
18	Do you have a long-term lung disease (such as chronic bronchitis or chronic obstructive pulmonary disease)?	98	Good
19	Do you have or have you had sleep apnoea?	92	Moderate
20	Have you been told that you snore so loud you keep others awake while you are asleep?	91	Moderate
21	Have you ever had an X-ray of your chest?	86	Poor
22	Do you smoke or have you ever smoked?	100	Good
23	Do you have gastric reflux or heartburn?	85	Poor
24	Do you have or have you ever had liver problems (such as hepatitis or cirrhosis)?	98	Good
25	How many days a week do you drink alcohol (on average)? ^a^	–	–
26	Do you have or have you ever had abnormal kidney function or kidney disease?	100	Good
27	Have you ever had a (minor) stroke or a brain bleed?	100	Good
28	Do you have or have you ever had fits/seizures/epilepsy?	99	Good
29	Have you ever lost consciousness?	99	Good
30	Do you have or have you ever had diabetes or diabetes related to pregnancy?	98	Good
31	Do you have or have you ever had thyroid problems (e.g. thyroid hormone levels being too high or too low or having an enlarged thyroid)?	93	Moderate
32	Do you have loose/chipped teeth, crowns, bridges, veneers or dentures?	94	Moderate
33	Do you have difficulty swallowing?	98	Good
34	Do you have difficulty opening your mouth wide?	97	Good
35	Do you have or have you ever had pain or stiffness in the lower back, neck or jaw?	82	Poor
36	Have you ever been told that you have had problems with anaesthetics in a previous operation, such as an abnormal reaction to anaesthesia or allergy to anaesthetics?	95	Good
37	Has any of your blood relatives ever had problems with anaesthetics in a previous operation?	96	Good
38	Do you have or have you ever had anxiety, depression or other emotional/psychiatric disorders?	95	Good
39	Do you have any other medical information that we should know about?	98	Good

^a^ This question required a free-text response and thus, was excluded from reliability testing

#### Study 3

Of 104 patients recruited, 98 (94.2%) patients completed the study. They were predominantly of Chinese (55/55.1%), Malay (18/18.4%) and Indian (6/6.1%) ethnicity. Notably, 88 (89.8%) patients were below 50 years old. Median (IQR) completion time to self-assessment on the digital application was 6.4 (4.8, 8.6) minutes. Table 5 shows the results of reliability testing of Form 3. Good criterion validity was obtained for 23 of 37 (62%) questions. Eight (22%) questions had moderate criterion validity while 6 (16%) questions had poor criterion validity. Total number of response pairs was 3626. Of these, 3405 were identical, giving a concordance rate of 93.9%. There were 133 (3.7%) discrepant contradictory responses and 88 (2.4%) discrepant non-contradictory responses, giving a total of 221 (6.1%) discrepant responses. The most common discrepant response pair was “yes/no*”* (89/2.5%), followed by “unsure/no “(76/2.1%), “no/yes*”* (44/1.2%), “unsure/yes*”* (11/0.3%) and “yes/unsure*”* (1/0.03%).

**Table 5:** Inter-rater Reliability Testing of Form 3

		^a^PA	Criterion validity
1	Do you have any allergies (to medicines, sticking plaster, iodine, latex, food, etc.)?	97	Good
2	As medicines and supplements can affect body functions and interact with anaesthetics, please list all the medicines (including traditional medicines and health supplements) you are currently taking on a regular or daily basis in the last 2 weeks. ^b^	–	–
3	Have you ever had an operation (including major dental surgery e.g. wisdom teeth extraction)?	89	Poor
4	Are you ever short of breath after walking up two flights of stairs or an overhead bridge?	76	Poor
5	Have you ever had an ECG (or electrocardiogram) and been told it was not normal?	86	Poor
6	Has a doctor ever told you, you have high blood pressure, also known as ‘hypertension’?	95	Good
7	Do you have, or have you ever had chest pain that you felt tight or heavy (not from coughing)?	91	Moderate
8	Have you ever had a heart attack?	100	Good
9	Do you have frequent swelling in both feet or both ankles?	92	Moderate
10	Do you have, or have you ever had treatment for problems with your heartbeat (too low, too fast, irregular)?	97	Good
11	Has a doctor ever told you they heard an abnormal sound (e.g. a click or a murmur) whilst listening to your heart?	97	Good
12	Do you have a cardiac pacemaker or an implanted cardioverter-defibrillator?	98	Good
13	Have you ever had heart surgery (valve or stent or bypass operation)?	100	Good
14	Do you have or have you ever had blood clots in legs or lungs?	98	Good
15	Have you ever had a blood transfusion?	100	Good
16	Do you have asthma or have you had asthma as a child?	95	Good
17	Do you currently have a cough lasting more than 8 weeks?	97	Good
18	Do you have a long-term lung disease (such as chronic bronchitis or chronic obstructive pulmonary disease)?	99	Good
19	Has anyone told you that you stop breathing of choke during your sleep – a condition also known as sleep apnoea?	99	Good
20	Have you been told that you snore so loud you keep others awake while you are asleep?	90	Moderate
21	Do you often feel tired, fatigued or sleepy during the daytime (tired enough that you could fall asleep while performing activities e.g. driving, waking, texting)?	96	Good
22	Do you smoke or have you ever smoked?	90	Moderate
23	Do you have gastric reflux or heartburn?	80	Poor
24	Do you have or have you ever had liver problems (such as hepatitis or cirrhosis)?	97	Good
25	How many days a week do you drink alcohol (on average)? ^b^	–	–
26	Do you have or have you ever had abnormal kidney function or kidney disease?	100	Good
27	Have you ever had a (minor) stroke or a brain bleed?	100	Good
28	Do you have or have you ever had fits/seizures/epilepsy?	100	Good
29	Have you ever lost consciousness?	98	Good
30	Do you have or have you ever had diabetes or diabetes related to pregnancy?	98	Good
31	Do you have or have you ever had thyroid problems (e.g. thyroid hormone levels being too high or too low or having an enlarged thyroid)?	95	Good
32	Do you have loose/chipped teeth, crowns, bridges, veneers or dentures?	91	Moderate
33	Do you have difficulty swallowing?	97	Good
34	Do you have difficulty opening your mouth wide?	98	Good
35	Do you have or have you ever had pain or stiffness in the lower back, neck or jaw?	81	Poor
36	Have you ever been told that you have had problems with anaesthetics in a previous operation, such as an abnormal reaction to anaesthesia or allergy to anaesthetics?	94	Moderate
37	Has any of your blood relatives ever had problems with anaesthetics in a previous operation?	91	Moderate
38	Do you have or have you ever had anxiety, depression or other emotional/psychiatric disorders?	94	Moderate
39	Do you have any other medical information that we should know about?	83	Poor

^a^ denotes Percentage of Agreement

^b^ This question required a free-text response and thus, was excluded from reliability testing

Based on these findings, the working group made further enhancements to the digital application (Supplementary Box 6). In summary, the “unsure*”* option was deleted to encourage commitment to a definitive response. Probing stems of questions were also added to specific domains to improve qualification of symptoms. Drop-down options of past surgeries, medications and allergies were updated to include more choices. These amendments led to the development of an improved digital version. The feasibility of its implementation was reported in a study published recently [17].

## Discussion

Using a robust, mixed-methods approach, the present study describes the development and validation of a patient-administered digital assessment application on a tablet device for the purpose of gathering medical history relevant to preanaesthesia assessment. The PreAnaesthesia Computerized health Assessment (PATCH) application is accepted by patients and reliable when compared with nurse-led assessment.

For health assessment instruments to have practical value, they should have reliability and validity. An example of content validity is face validity – the extent to which items are perceived to be relevant to the intended construct, while criterion validity is a dimension of reliability. Compared to published studies [10], the present study achieved > 90% criterion validity in 84% of questions in the digital prototype. The difference could be related to differences in subject characteristics, such as literacy and social factors, which result in different perception and interpretation of the questions [15].

In the analysis of responses between patient self-assessment and nurse assessment, discrepant contradictory response-pairs, such as ““no/yes*”* and “unsure/yes*”*, can be concerning as they suggest failure of the digital tool to detect an issue that is eventually uncovered by nurse assessment. Fortunately, these constitute only 3.7% of total responses in the validation of the digital prototype. The fact that 93.9% of total response-pairs were concordant strongly supports its reliability in gathering preoperative medical information.

We observed that participants in the present study were mainly English-literate female, with median age < 40 years. Studies suggest that age and literacy can affect a patient’s perception and willingness to adopt mobile health technology. In a systematic review to evaluate barriers in adopting telemedicine, age, level of education and computer literacy emerged as key patient-related determinants [18]. The authors speculated that preference for personalised care and lack of training in new technology among older patients could have contributed to this observation. In another study that examined the usage patterns of virtual health services, younger and predominantly female patients were more likely to be early adopters of virtual medical consultations [19]. In driving digital strategies for patient care, healthcare organisations must address the needs of patients and tailor the engagement platform according to patients’ prefences and technology know-how. In the present study, 83.7% of participants in Phase 2 of the study had expressed receptiveness to the use of a digital self- assessment tool. This is not surprising, given the young age of our patients and the high internet penetration rate in the local population [20]. Concerns of data breach should be addressed with strict regulation and compliance with Health Level 7 (HL7) standards, through secure networks, data encryption, login controls and auditing.

Positive patient acceptance of digital health assessment has motivated us to re-design our clinical pathways, leveraging on telemedicine to achieve greater value. PATCH could serve as an online triage tool to determine if patients undergo a tele-consultation or in-person consultation for anaesthetic referral. With an average patient wait-time of 24 min at the preoperative evaluation clinic (unpublished data from internal audit), conversion of physical to tele-consultation could improve patient experience and clinic efficiency. The clinic could, in turn, focus its resources on optimizing care for medically-complex patients who present physically for consultation. Reducing physical visits to healthcare facilities could also confer the benefit of reducing the transmission of infectious diseases [21].

There are limitations to the present study. Recruitment of a larger sample would have allowed the use of Kappa coefficient for measurement of criterion validity. As the patients’ socio-economic characteristics were not reported, we could not control for bias due to socio-economic factors. The study was conducted in young adult female patients of a local healthcare facility. The study may yield different results in a mixed gender population or another clinical setting. As the application is developed in English, the results may not be extrapolated to questionnaires translated to other languages. Further research is directed at improving validity of the digital application and adapting it for use in other languages. There is also a plan to incorporate decision support tools to aid in risk prediction and clinical decision-making. To maintain the human touch, questions would be developed to simulate human-to-human conversation, incorporating elements of empathy [22] – a technique demonstrated to evoke responses more effectively from subjects during a computerised interview.

## Conclusion

Self-administration of digitized preanaesthesia health assessment is acceptable to patients and reliable in eliciting medical history. Further iteration should focus on improving reliability of the digital tool, adapting it for use in other languages and incorporating clinical decision tools.

## Additional file

Additional file 1: **Supplementary Material**. (DOCX 270 kb)

## Data Availability

Data collected and analyzed for the study are available from the corresponding author upon reasonable request.

## Abbreviations

PATCH: Preanaesthesia computerized health (assessment)
PHA: Preanaesthesia health assessment
SDA: Same-day-admission
PA: Percentage agreement
