# Supplementary material for: PreAnaesThesia computerized health (PATCH) assessment: development and validation
Source: BMC Anesthesiol. 2020 Nov 14;20:286. doi: 10.1186/s12871-020-01202-8 (PMC7666442; doi:10.1186/s12871-020-01202-8)

**Supplementary Material**

| **Supplementary Box 1 : Form 1** |
| --- |
| Sex F/M |
| Weight ___ kg |
| Height ___ m |
| **General information** |
| Do you have any allergies (to medicines, sticking plaster, iodine, latex, food, etc.)? **^†^** |
| Please list all your medication to the best of your knowledge  (For example: Blood thinners, steroids, diabetic medications, medication bought without prescription, inhalers, creams/ointments, eye drops, pain relievers, herbal medication - this includes all other traditional medicines) |
| Name of medication |
| Dose |
| Using since |
| Have you ever had surgery? **^†^** |
| Year of surgery |
| Location (hospital/clinic) |
| What kind of operation? |
| **Questions about your heart and blood flow ^†^** |
| Do you have, or have you ever had chest pain that you felt tight or heavy (not from coughing)? |
| Have you ever had a heart attack? |
|  |
|  |
| Has a doctor ever told you they heard an abnormal sound (e.g. a click or a murmur) whilst listening to your heart? |
| Are you ever short of breath after walking up two flights of stairs or an overhead bridge? |
|  |
| Was your heart activity ever measured using wires on your chest (an ECG or electrocardiogram)? **^§^** |
| If Yes, have you been told that something was wrong? |
| Has a doctor ever told you, you have high blood pressure, also known as ‘hypertension’? |
| If Yes, are you taking medication for it? |
| Do you have, or have you ever had treatment for problems with your heartbeat (too low, too fast, irregular)? |
| Do you have frequent swelling in feet or ankles? |
| Do you have a cardiac pacemaker or an implanted cardioverter-defibrillator? |
| Have you ever had heart surgery (valve or stent or bypass operation)? |
| Do you have or have you ever had blood clots in legs or lungs? |
| Have you ever had a blood transfusion? |
| **Questions about your breathing and lungs ^†^** |
| Do you have a cough lasting more than 8 weeks? |
| Do you have asthma or have you had asthma as a child? **^§^** |
| If Yes, when? |
| If Yes, are/were you on inhalers or other medication? |
| If Yes, were you ever admitted to hospital? |
| Do you have a long-term lung disease (such as chronic bronchitis or chronic obstructive pulmonary disease)? |
| Do you have or have you had sleep apnea? |
|  |
| Have you been told that you snore so loud you keep others awake while you are asleep? |
| Have you ever had an X-ray of your chest? **^§^** |
| If Yes, have you been told that something was wrong? |
| Do you smoke or have you ever smoked? **^§^** |
| If Yes, ___ Sticks ___ Day ___Years |
| Still smoking/Stopped smoking since _______(date) |
| **Questions about your digestive and urinary systems ^†^** |
| Do you have or have you ever had liver problems (such as hepatitis or cirrhosis)? |
|  |
| Do you have gastric reflux or heartburn? |
| Do you have or have you ever had abnormal kidney function or kidney disease? |
| How many days a week do you drink alcohol (on average)? **^*^** |
| __________ Days |
| **Questions about your brain ^†^** |
| Have you ever had a (minor) stroke or a brainbleed? |
| Do you have or have you ever had fits/seizures/epilepsy? **^§^** |
| If Yes, have you been seizure-free for more than 6 months? |
| Have you ever lost consciousness? |
| **Questions about your hormones ^†^** |
| Do you have or have you ever had diabetes or diabetes related to pregnancy? |
|  |
| Do you have or have you ever had thyroid problems (hypo/hyper or an enlarged thyroid)? |
|  |
| **Questions about anaesthesia ^†^** |
| Do you have or have you ever had pain or stiffness in the lower back, neck or jaw? |
| Have you ever had problems with doctors putting you to sleep in previous operations? |
| Has any of your blood relatives ever had problems with doctors putting them to sleep in previous operations? |
| Do you have loose/chipped teeth, crowns, bridges, implants, veneers, dentures? |
|  |
|  |
|  |
| Do you have difficulty swallowing? |
| Do you have difficulty opening your mouth wide? |
| **Other questions ^†^** |
| Do you have or have you ever had anxiety, depression or other emotional/psychiatric disorders? |
| Do you have any other medical information that we should know about? |

| **Supplementary Box 2. Glossary of Terms**   1. **Allergies** are unexpected reactions from your body to certain products or food. After using or eating these you can get skin problems (redness, itching) or breathing problems. 2. A **cardiac pacemaker** or a **cardioverter-defibrillator** is a medical device implanted under your skin when you have problems with your heart rhythm. You would have been admitted to hospital to get this device implanted. 3. **Chronic bronchitis** or **chronic obstructive pulmonary disease** (COPD) are long-term lung diseases often caused by cigarette smoking. Symptoms are shortness of breath, exhaustion, coughing and phlegm. 4. **Sleep apnoea** is a disorder where your breathing pauses repeatedly or you have shallow breathing during your sleep. Symptoms are loud snoring when you are asleep, feeling exhausted when you wake up, waking up with a headache and sleepiness during the day. 5. A **chest X-ray** is used to take a ‘picture’ of your lungs. 6. **Hepatitis** is a disease of the liver, caused by inflammation or infection. There are a few types of hepatitis e.g. Hepatitis A, B, C and D. 7. **Kidney disease** occurs when a person’s kidney stop working properly or fail. 8. A **stroke** is caused by a blockage of blood flow or rupture of blood vessels in the head. After a stroke, people can have numbness and weakness of body parts or can have problems with thinking and remembering. 9. **Epilepsy** can cause abnormal shaking of the body. 10. **Anaesthetics** are medications used during surgery to make you sleep deeply and relax (called general anaesthesia) or to numb a particular part of the body (called regional anaesthesia). Anaesthetics allow people to undergo surgery without feeling pain |
| --- |

| **Supplementary Box 3. Amendments made to develop Form 2**   1. An image of an ECG was inserted alongside the question: Was your heart activity ever measured using wires on your chest (an ECG or electrocardiogram)? 2. An image of a pacemaker was inserted alongside the question: Do you have a cardiac pacemaker or an implanted cardioverter-defibrillator? 3. A question on height and weight was added. Allows for calculation of BMI and identification of patients with high BMI. BMI >37.5 is a criterion for anaesthetic review. ^1^ 4. Questions that were re-phrased:    1. Please list all your medication to the best of your knowledge       1. As medicines and supplements can affect body functions and interact with anaesthetics, please list all the medicines (including traditional medicines and health supplements) you are currently taking on a regular or daily basis in the last 2 weeks. ^2^    2. Have you ever had surgery?       1. Have you ever had an operation (including major dental surgery e.g. wisdom teeth extraction)?          1. Nature of operation          2. Location (hospital/clinic) ^3^          3. Year of operation ^4^    3. Do you have a cough lasting more than 8 weeks?       1. Do you currently have a cough lasting more than 8 weeks?    4. Do you have or have you ever had thyroid problems (hypo/hyper or an enlarged thyroid)?       1. Do you have or have you ever had thyroid problems (e.g. thyroid hormones being too high or too low or having an enlarged thyroid)?    5. Have you ever had problems with doctors putting you to sleep in previous operations?       1. Have you ever been told that you have had problems with anaesthetics in a previous operation, such as an abnormal reaction to anaesthesia or allergy to anaesthetics?    6. Has any of your blood relatives ever had problems with doctors putting them to sleep in previous operations?       1. Has any of your blood relatives ever had problems with anaesthetics in a previous operation? 5. Questions that were deleted:    1. Still smoking/Stopped smoking since _______(date) 6. Terms in *Glossary* that were added or re-worded to improve explanation 7. Anaesthetics are medications used during surgery to make you unconscious and relaxed (general anaesthesia) or to numb a particular part of the body so that you do not experience pain (regional anaesthesia). Anaesthetic drugs/Anaesthetics are medications used to induce the state of anaesthesia. 8. Sleep apnoea is a disorder whereby your breathing repeatedly stops and starts during sleep. Symptoms and signs are loud snoring with choking sensation during sleep, feeling exhausted upon waking up, morning headaches and sleepiness during the day. The diagnosis is usually confirmed through a sleep study. 9. A chest X-ray is an imaging test that uses very small amounts of radiation to produce a ‘picture’ of your lungs, heart and surrounding structures. 10. Gastric reflux is the movement or rise of acidic stomach contents back into the throat. This can produce an unpleasant burning sensation in the chest, neck or throat, called heartburn. 11. Cirrhosis is a serious disease of the liver, whereby the healthy liver is gradually replaced by scar tissue, resulting in progressive liver failure. It is often the end-point of long-term hepatitis or alcohol intake. 12. A stroke is caused by a lack of oxygen to the brain, resulting from either blockage of blood flow or rupture of blood vessels in the head. After a stroke, people can have numbness and weakness of body parts or can have problems with thinking and remembering. 13. Epilepsy is a condition caused by abnormal firing of brain cells (sudden, intense bursts of electrical activity in the brain), resulting in repeated episodes of abnormal jerking of the body. Some people may lose consciousness. 14. Blood transfusion is the process of receiving blood or blood components taken from another person (the donor) into your bloodstream. 15. Changed the opening statement:     1. The questions below are asked to check your health before you undergo your operation. Please answer the questions to the best of your knowledge. If you are not sure, please tick Unsure. The superscript numbers (^1,2^) refer to the added help-sheet with further information.        1. The questions below are asked to assess your anaesthetic risk before you undergo your operation. Please answer the questions to the best of your knowledge. If you are not sure, please tick **Unsure**. Please refer to the glossary sheet if you need further explanations on any terms used. |
| --- |

| **Supplementary Box 4. Amendments made to develop Form 3**   1. Questions that were re-phrased:    1. Was your heart activity ever measured using wires on your chest (an ECG or electrocardiogram)?       1. Have you ever had an ECG (or electrocardiogram) and been told that it was not normal?    2. Do you have frequent swelling in feet or ankles?       1. Do you have constant swelling of both feet or both ankles?    3. [Smoking] If Yes, ___ Sticks ___ Day ___Years       1. If Yes, ___ Sticks per day (on average), for ___Years 2. STOP-BANG questions (Original and modified in Form 3 or 4) (added to Form 4, modified from Form 3 or taken as they are)    1. Do you *s*nore loudly (louder than talking or loud enough to be heard through closed doors)?       1. Have you been told that you snore so loud you keep others awake while you are asleep? (in form 3 and left unchanged in form 4)    2. Do you often feel *t*ired, fatigued, or sleepy during daytime?    3. Do you often feel tired, fatigued or sleepy during the daytime (tired enough that you could fall asleep while performing activities e.g. driving, walking, texting)? (added to form 4)    4. Has anyone *o*bserved you stop breathing during your sleep?       1. Do you have or have you had sleep apnea? (in form 3)          1. Has anyone told you that you stop breathing or choke during your sleep – a condition known as sleep apnoea? (added to form 4)    5. Do you have or are you being treated for high blood *p*ressure?       1. Has a doctor ever told you, you have high blood pressure, also known as ‘hypertension’? (in form 3 and left unchanged in form 4)    6. BMI more than 35 kg/m2? (in form 3 calculated by nurse, but in form 4 computed by app)    7. Age over 50 years old?       1. What is your age? (added to form 4)          1. Less than 50 years          2. 50 years and above    8. Neck circumference greater than 40 cm?       1. Entered by nurse into desktop version. 3. Construction of drop-down list of response options for questions on medicines, operations and allergies. 4. “Extra information help sheet    1. Added a new definition for electrocardiogram (ECG): This is a recording of the electrical activity of the heart. Electrodes are placed on the skin of the chest and connected in a specific order to a machine that, when turned on, measures electrical activity of the heart.    2. Deleted the definition for chest x-ray. 5. Deleted “If yes, please explain” for the following questions    1. Have you ever had an ECG (or electrocardiogram) and been told that it was not normal?    2. Has a doctor ever told you, you have high blood pressure, also known as ‘hypertension’?    3. Do you have asthma or have you had asthma as a child? 6. Delete    1. Have you ever had an X-ray of your chest?       1. If Yes, have you been told that something was wrong? 7. Framework for referrel to anaesthetist based on the STOP-BANG score    1. if STOP-BANG score is 2 or less : low risk of OSA 🡪 no need to refer to anaesthetist    2. if STOP-BANG score is 5 or more: high risk for OSA 🡪 refer to anaesthetist    3. if STOP-BANG score is 3-4 : look at STOP score and BMI       1. if STOP score 2 or more and BMI < 35 kg/m2 : low risk for OSA 🡪 no need to refer to anaesthetist       2. if STOP score 2 or more and BMI is 35kg/m2 or more : high risk for OSA 🡪 refer to anaesthetist 8. Designed a summary output to enable patient to revise and edit assessment before submitting. 9. Designed an introductory statement    1. Hello! Welcome to KKH Pre-Admission Services. Please enter your queue number to get started! If you have previously saved this questionnaire today, please enter the same queue number to resume filling that questionnaire. 10. Designed an instructions statement     1. The following questions are asked to assess your anaesthetic risk before you undergo your operations. Please answer the questions to the best of your knowledge. If you are not sure, please select Unsure. Some questions will have blue text like this. Let’s start with some general questions. 11. Designed a closing statement     1. Thank you! Your completed questionnaire has been submitted to the clinic. The nurse will review your questionnaire shortly. Once your queue number is called, kindly proceed to the designated counter. |
| --- |

| **Supplementary Box 5. Amendments made to improve on Form 3**   1. Delete “Unsure” response option from the following questions:    1. Has a doctor ever told you, you have high blood pressure, also known as ‘hypertension’?    2. Have you ever had heart surgery (valve or stent or bypass operation)?    3. Do you have a cardiac pacemaker or an implanted cardioverter-defibrillator?    4. Have you ever had a heart attack?    5. Has anyone told you that you stop breathing or choke during your sleep – a condition known as sleep apnoea?    6. Have you been told that you snore so loud you keep others awake while you are asleep?    7. Do you often feel tired, fatigued or sleepy during the daytime (tired enough that you could fall asleep while performing activities e.g. driving, walking, texting)?    8. Do you smoke or have you ever smoked?    9. Do you have difficulty swallowing?    10. Do you have difficulty opening your mouth fully?    11. Do you have any other medical information that we should know about?    12. Do you suffer constant pain or stiffness of the lower back, neck or jaw? 2. Amendments were made to the following questions    1. Have you ever had an operation (including major dental surgery e.g. wisdom teeth extraction)?       1. Have you ever had an operation in which you received local or general anaesthesia?    2. Are you ever short of breath after walking up two flights of stairs or an overhead bridge?       1. Are you ever short of breath after walking up two flights of stairs without stopping?    3. Have you ever had an ECG (or electrocardiogram) and been told that it was not normal?       1. Have you ever had an ECG (or electrocardiogram)? If Yes, have you been told it was not normal?    4. Do you have asthma or have you had asthma as a child? If Yes, are/were you on inhalers or other medication?       1. Do you have asthma or have you had asthma as a child? If Yes, did you have an asthma attack within the last 3 months?    5. Do you have gastric reflux or heartburn?       1. Do you feel the rise of stomach contents back into your throat at least 2 times a week? This condition is also known as gastric reflux or heartburn.    6. Do you have or have you ever had fits/seizures/epilepsy? If Yes, have you been seizure-free for more than 6 months?       1. Do you have or have you ever had fits/seizures/epilepsy? If Yes, have you had a seizure-free within the last 6 months?    7. Do you have or have you ever had diabetes or diabetes related to pregnancy?       1. Do you have diabetes? If Yes, are you on diet control, oral medications or injected medications?    8. Do you have or have you ever had thyroid problems (e.g. thyroid hormones being too high or too low or having an enlarged thyroid)?       1. Have you ever been told you have thyroid hormone levels that are too high or too low? Have you ever been told you have a thyroid swelling or thyroid lump?    9. Do you have or have you ever had pain or stiffness in the lower back, neck or jaw?       1. Do you suffer constant pain or stiffness of the lower back, neck or jaw?    10. Have you ever been told that you have had problems with anaesthetics in a previous operation, such as an abnormal reaction to anaesthesia or allergy to anaesthetics?        1. Have you ever been told that you have had problems with anaesthetics in a previous operation or dental procedure, such as an abnormal reaction to anaesthesia or allergy to anaesthetics?    11. Has any of your blood relatives ever had problems with anaesthetics in a previous operation?        1. Has any of your blood-related parents or siblings ever had experienced abnormal reactions or delayed awakening from anaesthesia?    12. Do you have difficulty opening your mouth wide?        1. Do you have difficulty opening your mouth fully? 3. Deletion of the following main and follow-up questions.    1. Have you ever had a blood transfusion?    2. If Yes, are/were you on inhalers or other medication? (for question on asthma)    3. If Yes, were you ever admitted to hospital? 4. Drop-down lists    1. Updated drop-down list of medicines, operations and allergies. |
| --- |

Supplementary figure 1. Sample screenshots of PATCH prototype


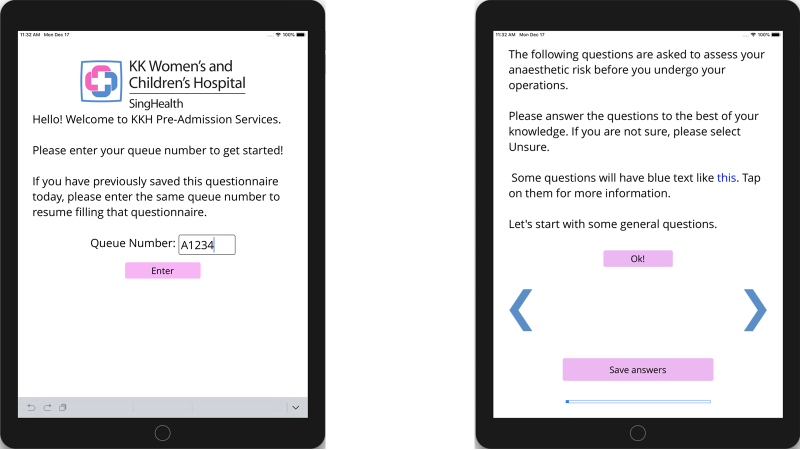


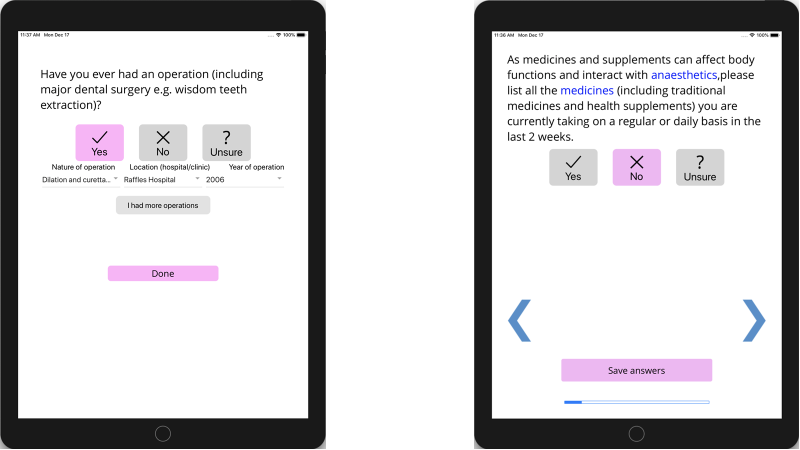


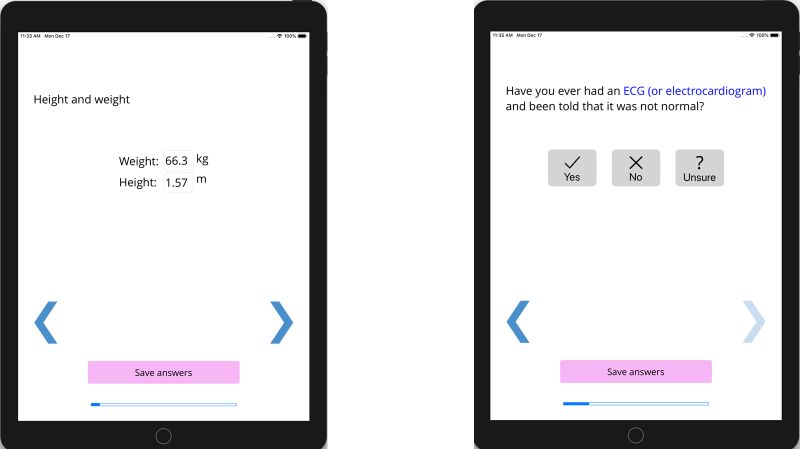


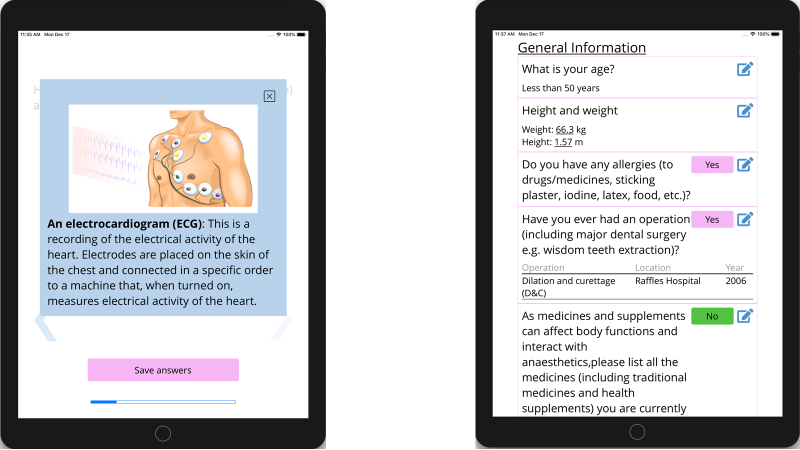

Supplement: Supplementary file 1 — Supplementary Material. (DOCX 270 kb) [file 12871_2020_1202_MOESM1_ESM.docx]
